# Motive Satisfaction in Chronic Pain Patients: Does It Improve in Multidisciplinary Inpatient Treatment and, if so, Does It Matter?

**DOI:** 10.1007/s10880-020-09718-4

**Published:** 2020-04-27

**Authors:** Alessia M. Vincent, Julian A. Stewart, Niklaus Egloff, Martin grosse Holtforth

**Affiliations:** 1 Department of Psychology, University of Bern, Bern, Switzerland; 2 Department of Medical Communication and Psychosomatic Medicine, Basel University Hospital, Basel, Switzerland; 3 Department of Psychosomatic Medicine, Department of Neurology, Inselspital, Bern University Hospital, Bern, Switzerland

**Keywords:** Chronic pain, Motivational incongruence, Motive satisfaction, Multimodal pain therapy, Psychological distress

## Abstract

According to consistency theory, insufficient motive satisfaction (motivational incongruence) is associated with psychological distress and mental disorders. High levels of distress and comorbid psychological disorders are common in patients with chronic pain. The aim of the present study was to investigate the role of motivational incongruence in chronic pain patients and the association of incongruence change with symptom improvement. Inpatients with chronic pain in multimodal interdisciplinary treatment (*n* = 177) completed questionnaires measuring motivational incongruence, psychological distress, pain intensity and pain interference at the beginning and end of a multimodal interdisciplinary inpatient treatment program at a tertiary psychosomatic university clinic. Results demonstrated that pain and motivational incongruence were significantly reduced at post-treatment, and reductions in incongruence were associated with reductions in psychological distress. In particular, better satisfaction of approach motives mediated the association between reduction of pain interference and psychological distress at post-treatment. Findings suggest that a reduction of motivational incongruence may be part of successful treatment of chronic pain.

## Introduction

Chronic pain is a highly prevalent condition that causes extensive suffering and disability (Johannes, Le, Zhou, Johnston, & Dworkin, 2010; Reid et al., 2011). According to the biopsychosocial model of chronic pain, biological, psychological and social factors play central roles in the development and chronification of pain (Turk & Okifuji, 2002). Of the psychosocial factors, dysfunctional cognitions and emotions (e.g., beliefs, lack of self-efficacy, fear avoidance), and various social variables (e.g., positive and negative social reinforcement by the environment, activities of daily living, family environment, cultural factors) have been identified as important contributors in this context (Gatchel, Peng, Peters, Fuchs, & Turk, 2007; Turk & Okifuji, 2002).

In *Consistency Theory*, Klaus Grawe (2004) formulated a comprehensive model for the understanding of mental disorders founded in basic psychological concepts as well as mechanisms of action in psychotherapy (Gassmann & Grawe, 2006; Smith & Grawe, 2003, 2005). This model was intended to inform psychotherapy practice and training and is to be continually updated by ongoing research (e.g., Gmeinwieser, Hagmayer, Pieh, & Probst, 2019; Mander, Jacob, Götz, Sammet, Zipfel, & Teufel, 2015; Silberschatz, 2017). Consistency theory assumes that individuals strive for “compatibility of many simultaneously transpiring mental processes” (Grawe, 2007, p.170), as well as *congruence* between their psychological needs (attachment, control, self-esteem enhancement, pleasure) and perceived reality (Caspar & grosse Holtforth, 2010; Grawe, 2004, 2007). Congruence between needs and perceived reality is assumed to be achieved by motive satisfaction via personal goals and related behaviors. Individual motives can be subdivided into approach and avoidance motives, in which approach motives serve to bring about desired experiences (need satisfaction), whereas avoidance motives serve to avert undesired experiences to protect one’s psychological needs from being hurt (Westermann, grosse Holtforth, & Michalak, 2019). Accordingly, the discrepancy between one’s motives and perceived reality has been coined as (*motivational) incongruence* (grosse Holtforth & Grawe, 2003; Westermann et al., 2019) or as insufficient motive satisfaction (Brockmeyer et al., 2014). Accordingly, a person experiences *approach incongruence* if perceived reality does not sufficiently satisfy one’s approach motives by pleasant experiences, and *avoidance incongruence* if the person cannot sufficiently avoid unpleasant experiences. According to consistency theory, congruence/motive satisfaction fosters adaptive functioning and psychological health, and incongruence is considered as a major source of psychological distress as well as a central risk factor for the development and maintenance of psychological disorders (Fries & Grawe, 2006). Psychological distress as well as mental and physical disorders are also potential sources of incongruence, considering the multiple real-life consequences mental and physical symptoms can have (Westermann et al., 2019).

Consistency theory can be considered an integrative theory of psychotherapy that chooses a motivational focus, i.e., is disorder-unspecific and may incorporate underlying approaches of disorder-specific change theories and related interventions (Grawe, 2004). For instance, cognitive-behavioral concepts such as fear avoidance (Lethem, Slade, Troup, & Bentley, 1983), or pain catastrophizing (Quartana, Campbell, & Edwards, 2009) can be understood in terms of consistency theory to both enhance incongruence between the goal of being pain-free and the actual perceived pain symptoms. Further, the underlying models in acceptance and commitment therapy (Hann & McCracken, 2014) may be interpreted as such that the acceptance of pain may reduce incongruence of the perceived pain. Also, consistency theory has some conceptual overlap with Young’s early maladaptive schemas (Mander et al., 2015).

Empirical evidence supports the association between incongruence, psychological distress and mental disorders. Psychotherapy patients have higher incongruence scores than healthy control groups (grosse Holtforth & Grawe, 2003; grosse Holtforth, Grawe, & Tamcan, 2004). Also, the level of incongruence can be reduced substantially through inpatient cognitive-behavioral therapy for different psychological disorders, and a reduction in incongruence after treatment is related to good psychotherapy outcomes (Berking, grosse Holtforth, & Jacobi, 2003). In addition, motivational incongruence has been found to relate to high levels of psychological symptoms such as anxiety and depression as well as low levels of well-being (Brockmeyer et al., 2014; Kelly, Mansell, & Wood, 2015). Particularly the attainment of approach goals, as opposed to avoidance goals, has been found to relate to symptomatic improvement (Wollburg & Braukhaus, 2010). In medical settings, incongruence has been linked to physical health. For example, incongruence predicted cardiovascular-related hospitalizations and all-cause mortality for patients with cardiovascular disease independent of traditional risk factors (Meyer, Stauber, Wilhelm, Znoj, & von Känel, 2015). In the workplace, incongruence was associated with job burnout and physical symptoms (Brandstätter, Job, & Schulze, 2016) and mediated the relationship between work-life balance and subjective well-being (Gröpel & Kuhl, 2009). Neurophysiologically, insufficient motive satisfaction was related to changes in resting state brain activity in healthy controls (Stein, Egenolf, Dierks, Caspar, & Koenig, 2013). Importantly, incongruence scores are partly independent of scores in other related self-report measures such as well-being (Berking et al., 2003). Regarding chronic pain, some research investigated the relationship between chronic pain and personal goal frustration, a concept similar to motivational incongruence. For instance, pain can interfere with personal goals regarding physical integrity or identity-related goals, which in turn may trigger emotional responses such as anger, frustration or guilt (Vervoort & Trost, 2017). However, to the best of our knowledge, there has been no targeted research on incongruence in chronic pain patients to date.

Taken together, there is ample evidence supporting associations between incongruence, psychological and physical distress. Thus, conceptualizing chronic pain in terms of incongruence may help to improve our understanding of chronic pain and its treatment by embedding it in a larger conceptual and therapeutic context.

With regard to patient functioning, research shows that patients with chronic pain may suffer from role loss in different social domains, and the number of roles and attributes lost due to their disability was found to predict depression scores (Harris, Morley, & Barton, 2003). This role loss may be explained by chronic pain patients frequently withdrawing from previously valued activities to avoid pain (Vlaeyen & Linton, 2012). Generally, disengaging from unattainable goals and reengaging in new, realistic goals seem to benefit well-being and quality of life (Esteve, Ramírez-Maestre, & López-Martínez, 2007; Van Damme, Crombez, & Eccleston, 2008; Viane et al., 2003). These findings suggest that chronic pain patients perceive incongruence regarding pain-related but also more general goals and that this incongruence may be associated with psychological distress. However, by disengaging from unattainable goals and redefining more realistic goals, chronic pain patients may reduce their perceived incongruence and foster psychological and physical well-being.

Extensive research has demonstrated associations between chronic pain and psychological distress. Depression has been found to precede, but also to be a consequence of chronic pain (Dersh, Polatin, & Gatchel, 2002) and a bidirectional influence of pain and depression in chronic pain patients has been demonstrated (Kroenke et al., 2011). Furthermore, pain, particularly in multiple locations, coincides with a higher risk for developing depressive and anxiety disorders later in life (Gerrits, van Oppen, van Marwijk, Penninx, & van der Horst, 2014). Other findings demonstrated temporal synchrony between pain and distress; higher depression and anxiety scores coincided with more severe pain and more pain locations, and a remission of depression or anxiety was associated with a significant reduction (Gerrits, van Marwijk, van Oppen, van der Horst, & Penninx, 2015). However, one study demonstrated that chronic pain does not predict future psychological distress, but rather that the presence of other physical and psychosocial factors in interaction with pain could lead to future distress (McBeth, Macfarlane, & Silman, 2002). Therefore, findings not only support associations between psychological distress and chronic pain, but also that other factors, such as incongruence, may play a role in this association. This warrants research investigating the link between chronic pain and psychological distress, chronic pain and goal frustration, and examination of how these variables are related.

The novelty and aim of this study were to investigate motivational incongruence in chronic pain patients before and after multimodal interdisciplinary chronic pain treatment. First, we hypothesize that chronic pain patients experience higher levels of incongruence overall than healthy subjects, following consistency theory’s assumptions that mental and physical symptoms are important sources of incongruence and that incongruence may be a risk factor for the development of psychopathological symptoms (grosse Holtforth & Grawe, 2003). Second, we hypothesize that a multimodal interdisciplinary treatment of chronic pain not only attenuates pain-related outcomes, but also reduces the level of experienced motivational incongruence. In an exploratory analysis, we will assess the change of the different incongruence dimensions over the course of treatment. Further, we will investigate the associations of incongruence reduction with general distress at post-treatment independent of other influence factors, as well as of approach incongruence, avoidance incongruence and single-item approach incongruence at post-treatment. Lastly, we will explore whether the reduction of motivational incongruence mediates the association between the reduction of pain interference and psychological distress at post-treatment.

## Methods

### Participants

Participants were inpatients with chronic pain in a multimodal interdisciplinary treatment program at a tertiary psychosomatic university clinic. On average, patients were 48.2 years old (*SD* = 14.1, range = 18–85), and 61.0% (*n* = 177) were women. The average duration of treatment was 22.6 days (*SD* = 2.80). In the analysis included were patients who fulfilled the diagnosis of chronic somatoform pain with somatic and psychological factors (F45.41) according to the ICD-10 (Dilling, Mombour, Schmidt, & Colart, 2015). These patients showed a mean illness duration of 4.39 years (SD = 1.32). Patients were excluded from the analysis if they were under the age of 18, did not have sufficient German language skills and did not complete the relevant questionnaires at pre- and/or post-treatment.

### Procedures

Between 2015 and 2018, data were collected from inpatients at the intake and discharge of the treatment program as part of routine quality assurance monitoring. Demographic information and clinical measures were assessed assisted by trained research assistants.

### Description of Treatment

The multimodal interdisciplinary therapy for chronic pain included medical interventions, pharmacotherapy, physiotherapy, occupational therapy and psychotherapeutic interventions. On average, patients participated in the treatment program for three weeks (*M* = 22.6 days, *SD* = 2.80 days).

### Measures

All patients completed a set of standardized self-report measures. The choice of outcome measures was informed by the IMMPACT recommendations, in which core outcome measures of chronic pain treatment include pain intensity, global rating of improvement, physical functioning, and emotional functioning (Dworkin et al., 2005). Sociodemographic data were assessed at pre-treatment. The following questionnaires were administered as part of a larger battery at pre- and post-treatment:

The Brief Pain Inventory (BPI; Cleeland & Ryan, 1994) measures both the severity of pain and interference of pain in the patient’s life and is used in its validated German version (Radbruch et al., 1999). Pain severity is calculated from four items on the intensity of pain, ranging from 0, no pain, to 10, the most severe pain imaginable. Pain interference is calculated with seven items which can be rated from 0, does not interfere, to 10, completely interferes. Both subscales add the corresponding items and average them, so that pain severity and pain interference can range between 0 and 10. The BPI is a frequently used generic pain questionnaire for different chronic pain conditions (Nolwenn & Lin, 2016).

The validated German short version of the Incongruence Questionnaire (INC-S; grosse Holtforth et al., 2004) was used to assess incongruence between a subject’s motives and perceived reality. The short version of the INC was constructed by selecting the item with the highest item-total correlations from each of the 23 subscales of the original 94-item version of the INC, the INC-S showing strong correlations with the INC questionnaire of *r* = .92–.98 tested in different patient populations (grosse Holtforth et al., 2004). The INC-S has two subscales, i.e., approach and avoidance incongruence. The 14 approach-incongruence items ask how strongly the individual currently experiences specific motive-satisfying transactions (e.g., ‘recently, I’ve had many social contacts’) and are rated on 5-point Likert scales ranging from 1 (high incongruence) to 5 (low incongruence). The 9 avoidance-incongruence items ask how strongly the individual currently experiences specific aversive transactions (e.g., ‘recently, I’ve felt helpless’) and are rated on 5-point Likert scales ranging from 1 (low incongruence) to 5 (high incongruence). The approach-incongruence score is computed as the average of the reversed approach-item ratings, the avoidance-incongruence score is calculated as the average of the avoidance-item ratings, and the total incongruence score is computed as the mean of the approach and avoidance-incongruence scores.

We used norm data of the INC-S (grosse Holtforth et al., 2004) to compare these incongruence values with those of our study’s chronic pain patient sample. This normal-control sample consisted of 707 participants (60.9% female) with an average age of 40.2 years (*SD* = 15.1; range = 18–87) neither having a diagnosed psychological disorder nor receiving psychological treatment.

The validated German version of the Hospital Anxiety and Depression Scale (HADS-D; Herrmann-Lingen, Buss, & Snaith, 2011) was used to assess anxiety, depression and general psychological distress. The questionnaire was developed specifically for use with patients with physical diseases or possibly psychogenic ailments and assesses the degree of anxious and depressive symptoms during the past week on two subscales with seven items each (Herrmann-Lingen et al., 2011). Items predominantly associated with physical symptoms are intentionally excluded, not to confound the assessment of psychopathological symptoms by likely somatic complaints. The HADS is highly recommended for the assessment of anxiety and depression in chronic pain populations (Pincus, Fraser, & Pearce, 1998). The sum of the two subscales can be used as a measure for general distress. Each item is scored from 0 to 3, leading to a possible score of 0 to 21 for each subscale and a score of 0 to 42 for the total score.

### Statistical Analyses

SPSS version 25.0 (SPSS Inc., Chicago, IL) was used for statistical analyses. The significance level was set at *p* < .05 (two-tailed), and list-wise comparisons were used for all statistical analyses. First, we computed descriptive statistics and Pearson-correlation coefficients between the outcome variables at pre- and post-treatment for chronic pain patients. Second, one-sample *t *tests were conducted to compare the incongruence subscales of the INC-S between chronic pain patients at intake and representative norms. Third, dependent sample *t *tests were conducted to test for differences between pre- and post-treatment scores. Differences in the subcategories of motivational incongruence between pre- and post-treatment were examined using *t* tests. Cohen’s *d* effect sizes were calculated for all *t* tests. Fourth, we used multiple regression analyses to examine the predictive value of change in motivational incongruence throughout the course of treatment on psychological distress at post-treatment above and beyond effects of the control variables age, gender, illness duration, and pain-related factors. The variance inflation factor (VIF) was computed to test for multicollinearity of the regression variables. Cross-lagged analysis were used to test for the likely causality of the regression analyses by testing the association of motivational incongruence with the change in psychological distress from pre- to post-treatment, above and beyond control variables and pain-related factors as in the previous analysis. As an exploratory analysis, a linear regression analysis with a stepwise elimination strategy including the same control variables and pain-related factors was used to examine the predictive value of the single-item approach incongruence on psychological distress at post-treatment. Last, we will consider relevant factors of the previous regression analyses to test a final mediation model in which we investigate the change of motivational incongruence throughout treatment as a mediator of the effect of pain interference on distress. To test the mediation, the PROCESS v3.0 script for SPSS Statistics 25.0 (IBM Corp.; Armonk, NY; USA) developed by Hayes (2013) was used. In the mediation analysis, we controlled for age, gender, illness duration and general distress at pre-treatment as covariates. Then, the reduction of pain interference was regressed on change of approach incongruence throughout treatment as the mediator and the outcome variable distress at post-treatment, and change of approach incongruence was regressed on distress at post-treatment. The indirect effect was tested using a bootstrap estimation approach for percentile bootstrap confidence intervals with 5000 samples and a level of confidence for all confidence intervals of 95%. To calculate *β, z*-standardized variables were calculated. Post hoc power analyses were conducted with G*Power 3.1 at an alpha level of *α* = .05 and our sample size of *n* = 177. For our pre–post analyses, the post hoc power analyses for *t* test dependent means demonstrates that an effect size of *d* = .3 can be shown with a power of 98%, and an effect size of *d* = .2 can be detected with a power of 75%. For our *t* test comparing our sample (*n* = 177) with a healthy norm sample (*n* = 707), post hoc power analyses showed a power of 94% for *d* = .3. For multiple regressions with 8 predictors, post hoc power analyses show a power of 100% for an *R*^2^ of .67. Lastly, for our mediation analyses, the app MedPower (Kenny, 2017) yielded a power of 87% for the detection of the indirect effect of *β* = .24.

## Results

### Pre-treatment Analysis

The means, standard deviations and Pearson correlations between the study variables are presented in Table 1. The high correlation of *r* = .68 between approach incongruence and avoidance incongruence at intake justified additionally calculating a total incongruence score as the average of approach incongruence and avoidance incongruence. Table 2 shows *t* tests comparing means between pain patients at pre-treatment and a norm sample of all incongruence items, as well as the average scores of approach incongruence, avoidance incongruence and overall motivational incongruence. As each INC-S item represents the corresponding scale of the long INC version on the basis of maximal item-total correlation (grosse Holtforth et al., 2004), respectively, the INC-S items were regarded as proxies of the INC scales. All incongruence scores and all INC-S items except for Appreciation/Approval, and Blame/Criticism, differed significantly between the two groups, ranging mostly between medium to high effect sizes. Chronic pain patients showed significantly higher incongruence scores than normal controls in most items. However, healthy control subjects showed higher incongruence values regarding Altruism, Receiving Help, and Hurting Others.

**Table 1:** Means, standard deviations and Pearson correlations between the study variables

	*M*	*SD*	1	2	3	4	5	6	7	8	9	10	11
1. Total incongruence pre-treatment	2.67	.71	–										
2. Approach incongruence pre-treatment	2.77	.81	.96***	–									
3. Avoidance incongruence pre-treatment	2.50	.72	.86***	.68***	–								
4. General distress pre-treatment	2.18	8.32	.64***	.62***	.53***	–							
5. Pain intensity pre-treatment	5.45	1.78	−.03	−.07	.04	.09	–						
6. Pain interference pre-treatment	5.86	1.88	.43***	.38***	.42***	.50***	.43***	–					
7. Total incongruence post-treatment	2.37	.73	.67***	.63***	.60***	.52***	.14	.43***	–				
8. Approach incongruence post-treatment	2.48	.83	.63***	.64***	.50***	.50***	.13	.40***	.95***	–			
9. Avoidance incongruence post-treatment	2.20	.74	.59***	.48***	.65***	.42***	.12	.39***	.85***	.65***	–		
10. General distress post-treatment	15.41	8.33	.58***	.53***	.54***	.72***	.25**	.49***	.74***	.69***	.64***	–	
11. Pain intensity post-treatment	5.10	2.01	.06	.01	.13	.14	.78***	.40***	.26***	.22**	.27***	.38***	–
12. Pain interference post-treatment	4.67	2.19	.35***	.28***	.39***	.36***	.46***	.62***	.59***	.55***	.54***	.65***	.62***

*N* = 177

***p < .001; **p < .01; *p < .05

**Table 2:** One-sample *t* test comparing means between pain patients at pre-treatment and a norm sample for all items of the INC-S

	Chronic pain patients pre-treatment		Healthy control group		*T*	Cohen's *d*
	*M*	*SD*	*M*	*SD*		
Intimacy/Attachment	2.91	1.65	2.46	1.18	3.62***	.31***
Affiliation/Sociability	3.02	1.49	2.46	1.01	4.98***	.44***
Altruism	2.06	1.25	2.37	.84	− 3.35**	.29**
Receiving Help	2.15	1.24	2.5	.85	− 3.73***	.33***
Appreciation/Approval	1.99	1.15	2.03	.66	− .48	.04
Status	2.95	1.39	2.64	.76	3.01**	.28**
Autonomy	2.89	1.46	2.05	.77	7.64***	.72***
Achievement/Performance	3.67	1.26	2.21	.78	15.44***	1.39***
Control	2.50	1.33	2.22	.74	2.83**	.26**
Education/Understanding	3.05	1.42	2.37	.70	6.38***	.61***
Belief/Sense of Meaning	2.65	1.39	2.26	.80	3.73***	.34***
Excitement/Diversion	3.39	1.39	2.33	.85	10.14***	.92***
Trust in Oneself	2.77	1.37	2.1	.78	6.48***	.60***
Self-Reward	2.85	1.44	2.5	.9	3.21**	.29**
Separation/Being Alone	2.20	1.36	1.99	.86	2.09*	.19*
Not Being Respected/Accepted	2.11	1.23	1.67	.72	4.72***	.43***
Humiliation/Embarrassment	2.06	1.29	1.53	.60	5.48***	.53***
Blame/Criticism	2.17	1.19	2.04	.86	1.45	.12
Dependence/Loss of Autonomy	2.51	1.37	1.88	.76	6.10***	.57***
Hurting Others	1.78	1.12	1.97	.76	− 2.25*	.20*
Weakness/Loss of Control	3.28	1.39	2.59	.82	6.58***	.60***
Helplessness	3.27	1.40	2.04	.87	11.69***	1.05***
Failure	3.16	1.34	1.82	.74	13.30***	1.24***
Approach Incongruence	2.77	.81	2.32	.54	7.44***	.65***
Avoidance Incongruence	2.50	.72	1.95	.59	10.21***	.84***
Total Incongruence	2.67	.72	2.13	.51	10.00***	.87***

*N*1 = 177; *N*2 = 707; *df* = 176

***p < .001; **p < .01; *p < .05

### Comparisons Between Pre- and Post-Treatment

**Fig. 1:**
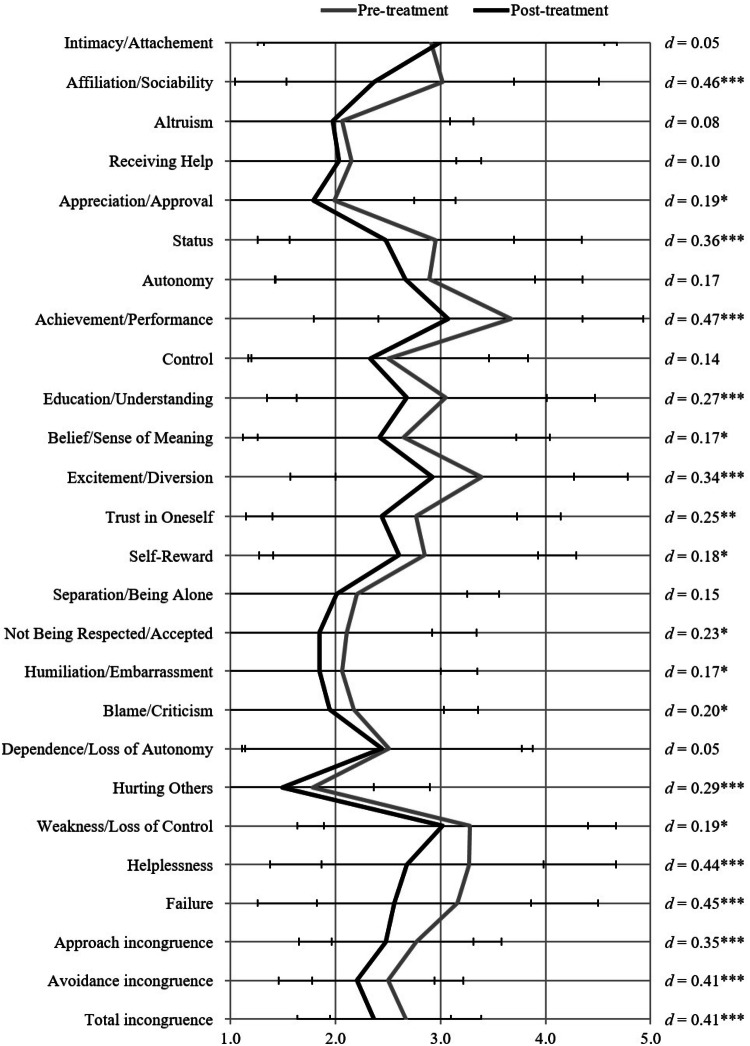
Means, standard deviations and Cohen’s d effect sizes of the INC-S items, subscales and total score at pre- and post-treatment

Table 3 shows the dependent *t* tests that were calculated for different outcome measures between pre- and post-treatment. All outcome measures significantly improved over the course of treatment. *T* tests exploring pre- and post-differences in all INC-S items are shown in Fig. 1. Patients reported lower incongruence at post-treatment in 16 of 23 domains, yet with considerable variation in significant effect sizes (*d* = .17–.47 for approach motives, *d* = .17–.45 for avoidance motives).

**Table 3:** Dependent *t* tests of multiple outcome variables between pre- and post-treatment

	Pre		Post		*T*	Cohen's *d*
	*M*	*SD*	*M*	*SD*		
Pain intensity	5.45	1.78	5.10	2.01	3.76***	.18***
Pain interference	5.86	1.88	4.67	2.19	8.82***	.58***
General distress	20.18	8.32	15.41	8.33	10.11***	.57***
Total incongruence	2.67	.72	2.37	.73	6.73***	.41***
Approach incongruence	2.77	.81	2.48	.83	5.52***	.35***
Avoidance incongruence	2.50	.72	2.20	.74	6.55***	.41***

*N* = 177; *df* = 176

***p < .001; **p < .01; *p < .05

### Multiple Regression Analysis

We first tested the association of general distress at post-treatment and the change of motivational incongruence throughout treatment, differentiating between change in approach and avoidance incongruence, above and beyond control variables and pain-related factors in a multiple regression analysis as shown in Table 4. The regression was statistically significant, *F*(6, 168) = 46.06, *p* < .001 *R*^2^ = .69, with an adjusted *R*^2^ of .67. Above and beyond control variables, general distress at intake, and change in pain variables, a decrease in incongruence in approach motives (i.e., better motive satisfaction) was associated with lower general distress at post-treatment, whereas the change of incongruence in avoidance goals did not.

**Table 4:** Multiple regression analysis with mean general distress at post-treatment assessment as outcome

	*B*	*SE*	*β*	*t*
Age	− .04	.03	− .07	− 1.47
Gender	− .56	.75	− .03	− .75
Illness duration	.57*	.28	.09*	2.05*
General distress pre-treatment	.75***	.04	.75***	16.97***
Pain intensity change score	.37	.32	.06	1.15
Pain interference change score	1.01***	.27	.22***	4.29***
Approach incongruence change score	2.14**	.62	.18**	3.46**
Avoidance incongruence change score	1.10	.71	.08	1.56

*N* = 177

***p < .001; **p < .01; *p < .05

The regression model was tested for multicollinearity. The variance inflation factor (VIF) showed that change of incongruence in approach goals, *VIF* = 1.47, tolerance = .68, and change of incongruence in avoidance goals, *VIF* = 1.44, tolerance = .70, were the strongest predictors in the model. Following guidelines by Menard (1995), Bowerman and O’Connell (1990) and Myers (1990), multicollinearity was unproblematic in this model. Given that both outcomes (distress change) and predictors (pain and incongruence change) were assessed at the same time points (pre- and post-treatment), cross-lagged analyses were computed to test whether the regression model may be interpreted approximately causal. For this purpose, a model was tested in which general incongruence was associated above and beyond control variables and pain-related factors in a multiple hierarchical regression analyses (Table 5). The regression was statistically significant, *F*(7, 169) = 38.15, *p* < .001 *R*^2^ = .61, with an adjusted *R*^2^ of .60. Due to this significant association of motivational incongruence at post-treatment by the change of general distress over treatment, the initial regression model cannot be understood causally, as both regression models reach significance. It rather proposes that motivational incongruence and general distress mutually influence each other, above and beyond control variables and pain-related factors.

**Table 5:** Multiple regression analysis with mean total incongruence at post-treatment assessment as outcome

	*B*	*SE*	*β*	*t*
Age	.01*	.01	.11*	2.21*
Gender	.04	.07	.03	.61
Illness duration	.02	.03	.04	.86
Total incongruence pre-treatment	.73***	.05	.72***	14.50***
Pain intensity change score	.01	.03	.02	.41
Pain interference change score	.06**	.02	.16**	2.73**
General distress change score	.03***	.01	.27***	4.80***

*N* = 177

***p < .001; **p < .01; *p < .05

As an exploratory analysis, we aimed at identifying approach motives, the satisfaction of which was associated with the best treatment outcomes in terms of distress reduction in our sample and setting. For this, we repeated the regression analysis reported in Table 4, but replaced the change scores of approach and avoidance incongruence by the change scores of the 14 single approach-incongruence items. We used a stepwise elimination strategy to identify the most predictive items. The first step with age, gender and illness duration was significant, *F*(3, 173) = 2.67, *p* < .05. The second step included general distress at pre-treatment, change in pain intensity and change in pain interference over the course of treatment and was significant, *F*(6, 170) = 51.26, *p* < .001, the additional outcome variance explained being 60.0%, *R*^2^ = .64, *F*_change_ (3, 170) = 95.47, resulting in an adjusted *R*^2^ of .63. The third step further included the change score of Belief/Sense of Meaning and also showed significance, *F*(7, 169) = 48.38, *p* < .01, with an additional explained variance of .02%, *R*^2^ = .67, *F*_change_ (1, 169) = 11.73, and an adjusted *R*^2^ of .65. The fourth step then included the change score of Self-Reward which was also significant, *F*(8, 168) = 44.69, *p* < .01, the additional explained variance being .01%, *R*^2^ = .68, *F*_change_ (1, 168) = 6.91, resulting in an adjusted *R*^2^ of .67. Lastly, the fifth step further included the change score of Control and was significant, *F*(9, 167) = 40.90, *p* < .05, with an additional explained variance of .01%, *R*^2^ = .69, *F*_change_ (1, 167) = 4.09, and an adjusted *R*^2^ of .67. The final analysis shown in Table 6 indicates that the increases in Belief/Sense of Meaning, Self-Reward and Control, i.e., a reduction of incongruence in those items, over the course of treatment were significantly associated with general distress as treatment outcome. Multicollinearity was unproblematic in this model (Bowerman & O’Connell, 1990; Menard, 1995; Myers, 1990). To correct for multiple comparison, Bonferroni correction was applied separately. Considering the 20 items in the explorative regression analysis, the adjusted *p* < .0025 marks statistical significance. After correcting for Bonferroni, only general distress at pre-treatment, *p* < .001, and change in pain interference, *p* < .001, reach statistical significance.

**Table 6:** Linear regression analysis with stepwise elimination with mean general distress at post-treatment assessment as outcome without Bonferroni correction

	*B*	*SE*	*β*	*t*
Control variables				
Age	− .03	.03	− .05	− 1.18
Gender	− .43	.76	− .03	− .57
Illness duration	.54	.29	.09	1.90
General distress pre-treatment	.74***	.04	.74***	16.62***
Pain intensity change score	.42	.32	.06	1.31
Pain interference change score	1.08***	.24	.23***	4.53***
Belief/sense of meaning change score	.64*	.28	.11*	2.28*
Self-reward change score	.69*	.28	.12*	2.50*
Control change score	.59*	.29	.10*	2.02**

*N* = 177

***p < .001; **p < .01; *p < .05

### Mediation Analyses

**Fig. 2:**
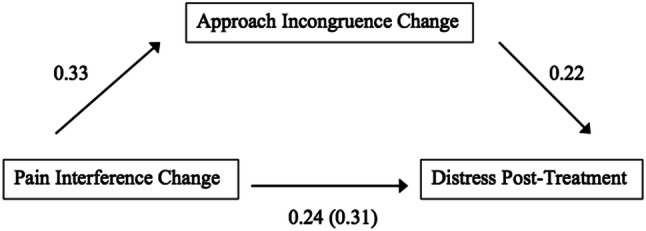
Mediation model of change in approach incongruence mediating the association between change in pain interference and general distress at post-treatment with z-standardized β-coefficients

A final mediation model considering relevant factors in the previous analyses was calculated to test if the change in approach incongruence throughout treatment mediates the effect of change in pain interference on general distress at post-treatment controlling for age, gender, illness duration and general distress at pre-treatment as covariates as shown in Fig. 2. In a first step, the relationship between pain-interference change and general distress was examined. Results indicated that pain-interference change was a significant predictor of general distress *β* = .31, *t*(171) = 6.95, *p* < .001, in an overall significant regression, *F*(5,171) = 61.20, *p* < .001, *R*^2^= .64. This indicates that individuals who experienced a decrease of pain interference throughout treatment also experienced less general distress at the end of treatment. The indirect effect was tested using a percentile bootstrap estimation approach with 5000 samples. These results showed that the indirect coefficient was significant (*β* = .07, *SE* = .12) with a 95% bootstrap confidence interval of .15 to .60, further indicating a significant mediation. Approximately 68% of the variance in general distress was accounted for by the predictors (*F*(6,170) = 59.81* p* < .001). Thus, there is a mediation between pain-interference change and general distress post-treatment by the change of approach incongruence throughout treatment.

## Discussion

The aim of the present study was to investigate motivational incongruence of inpatients in a multimodal interdisciplinary chronic pain treatment. First, the chronic pain patients reported higher levels of incongruence compared to healthy norms in all summary scores (approach, avoidance, and total) as well as in almost all one-item measures of incongruence domains, but with considerable variation in effect sizes (medium to high). Pain intensity, pain interference, psychological distress and motivational incongruence improved significantly from pre- to post-treatment. Whereas the reduction in approach-motive incongruence was associated with the reduction of general distress at post-treatment above and beyond improvements in pain-related factors, the reduction in avoidance-motive incongruence did not explain further outcome variance in psychological distress. However in reverse, reduced general distress was also correlated with change of incongruence significantly, so that these pre-post results do not favor any causal direction. Further, the change in approach incongruence (i.e., better satisfaction of approach motives) throughout treatment mediated the effect of change in pain interference on general distress at post-treatment.

Overall, chronic pain patients demonstrated a higher levels of motivational incongruence at the beginning of treatment compared to healthy norms, suggesting that insufficient satisfaction of motives may be a clinically relevant characteristic worth considering in this population. At the level of single incongruence domains, healthy subjects reported higher incongruence than chronic pain patients only regarding Altruism, Receiving Help and Hurting Others. This may be due to the patients receiving help and support from clinical staff, but also from fellow patients from intake on.

Of the single domains of motivational incongruence, the large majority (16 of 23 items) of the representative single-item measures improved from pre- to post-treatment, mostly with small to medium effect sizes. Some improvements may be attributed to therapeutic interventions as well as the social setting of inpatient therapy, such as improvements regarding Education/Understanding, Trust in Oneself, Belief/Sense of Meaning, and Self-Reward, as well as Affiliation/Sociability and Appreciation/Approval. Better motive satisfaction regarding perceived Status, Performance, Respect and less perceived Humiliation, Criticism, Weakness, Helplessness and Failure may reflect improvements regarding the patients’ sense of identity and social role over the course treatment. As pain has been shown to interfere also with personal identity-related goals (Vervoort & Trost, 2017), pain likely diminishes the degree of motive satisfaction and decreases incongruence levels. Furthermore, research has demonstrated that disengaging from unattainable goals and reengaging in new, realistic goals may benefit one’s well-being and quality of life (Esteve et al., 2007; Van Damme et al., 2008; Viane et al., 2003). Therefore, it might be possible that these reduced levels of incongruence are not only due to improved goal achievement but may also have occurred due to patients beginning to detach from old motives, goals or social roles and starting to engage in new, realistic goals. Potentially, patients learn to better integrate pain in their lives in a way that impairs their sense of identity to a lesser degree. A closer examination of predictors correlates and consequences of reduced incongruence seem promising and awaits further research.

On the basis of consistency theory’s empirically supported assumption that incongruence is a major contributor to the development and maintenance of psychopathological symptoms and distress (Berking et al., 2003; Brockmeyer et al., 2014), we tested for the first time in chronic pain patients whether changes in motivational incongruence over the course of therapy were associated with reductions in psychological distress at post-treatment. Results demonstrated that the reduction in approach-motive incongruence, i.e., better motive satisfaction, was correlated with less general distress at post-treatment above and beyond improvements in pain-related factors, whereas reductions in avoidance-motive incongruence did not add further explained variance in the model, these results being in line with previous research (Wollburg & Braukhaus, 2010). However, these effects based on pre-post assessments cannot be interpreted as causal due to the non-experimental design of the study and due to the significant findings of the cross-lagged analysis for the reversed direction of prediction, i.e., change of general distress being significantly correlated with reduced motivational incongruence at post-treatment. Bidirectional effects between incongruence and distress demonstrated in the present study support consistency theory’s biopsychosocial assumption that somatic, mental and social symptoms and distress can be major sources of incongruence (grosse Holtforth & Grawe, 2003). Therefore, mutual associations between reductions of incongruence and distress were demonstrated in this chronic pain patient sample.

When specifically focusing on the single items of approach goals, the increases in Belief/Sense of Meaning, Self-Reward and Control over the course of treatment were associated with psychological distress as treatment outcome. Yet, after correcting for multiple testing with Bonferroni, none of the single items of approach goals reached statistical significance. Nevertheless, due to the nature of this explorative research question and the inclusion of all 14 approach-incongruence goals, these results give a first indication to which issues merit special attention in therapy and may therefore be interpreted cautiously.

As we have demonstrated in the previous analyses, relevant factors associated with general distress at the end of multimodal treatment are change in pain interference and change in approach incongruence throughout treatment. Therefore, we attempted a final mediation model in which we tested whether change in approach incongruence mediated the relationship between change in pain interference throughout treatment and general distress at post-treatment. Results demonstrated a partial mediation between pain-interference change and psychological distress through change in approach incongruence. Therefore, a reduction of psychological distress at post-treatment may be partly explained by the reduction of approach incongruence. In other words and in accord with the assumptions by consistency theory (Grawe, 1998), chronic pain patients’ reduction in distress after a multimodal interdisciplinary treatment may be due to the increase in satisfaction of psychological needs via the satisfaction of approach motives. Furthermore, our findings correspond with research indicating that while psychopathological symptoms coincide with pain symptoms (Gerrits et al., 2015), psychological distress may be associated more strongly with the additional presence of other physical and psychosocial factors than chronic pain alone (McBeth et al., 2002). The present findings suggest that the reduction in chronic pain patients’ distress after inpatient treatment seems to depend on better functioning in life, and that better functioning seems to be, in part, experienced by patients as better being able to control their lives and to engage in rewarding activities, as well as by considering their lives as being more meaningful again.

### Clinical Implications, Limitations and Future Research

For clinical practice, our findings have several potential implications. First, the significant differences in incongruence levels between chronic pain patients and normal controls show that motivational incongruence seems to be a relevant factor for psychopathology and the treatment of chronic pain patients. Second, the differential association of inpatient treatment outcome by approach and avoidance incongruence suggests that an improvement of approach-motive satisfaction may be more important for treatment outcome than a reduction of avoidance motive-violating aversive experiences regarding distress reduction. This suggests that multimodal inpatient treatment should specifically focus on ways to increase motive satisfaction, more than focusing on reducing avoidance-motive incongruence. The exploratory regression analysis indicates several specific motive-satisfaction domains, which may be associated with improved treatment outcomes, i.e., the increase in Belief/Sense of Meaning, Self-Reward and Control. These exploratory results may suggest that interventions targeting these aspects may be most promising for a short-term multimodal inpatient treatment for chronic pain.

Several limitations of this study are worth mentioning. First, many participants were excluded due to missing data, primarily due to random time restrictions for assessment in a short-term inpatient treatment. Second, the present study’s design does not allow for any causal conclusions. In the routine-care setting, not all potentially confounding variables or variables of interest can be controlled for or specifically manipulated, since the primary goal in this setting is the successful treatment of chronic pain patients. Furthermore, the nature of multimodal interdisciplinary pain treatment in itself makes it difficult to control or manipulate specific variables, due to the fact that this treatment includes different forms of therapy and not all participants might receive the exact same treatment due to individual treatment tailoring. Herein, our goal was to capture if and how treatment outcome can be in part explained by the degree of patients’ motive satisfaction regardless of the specific therapy. Therefore, it cannot be ruled out that the different components of treatment could have had different effects on the incongruence and pain outcomes. Further, the predictive value of this model is limited due to this routine-care setting resulting in only pre–post analyses. Therefore, longitudinal studies should be used to be able to make valid predictive statements on the relationship between motivational incongruence and chronic pain.

Considering the found associations between motivational incongruence, pain interference and psychological distress in chronic pain patients at pre- and post-treatment, future research should continue to investigate motivational incongruence in chronic pain patients. Because this is the first study examining the association between motivational incongruence and chronic pain, results should not be generalized yet, and more studies on motivational incongruence in the field of chronic pain are necessary to be able to make any concluding statements. However, based on the theory of Grawe (1998), there is reason to believe that the present results could be replicated in other studies. To be able to make any conclusions on causality, controlled and experimental designs are needed.

Further, it is yet unclear which role pain chronification might play in the associations between incongruence, pain and distress. Pain chronification was found to be linked to treatment resistance (Borsook, Youssef, Simons, Elman, & Eccleston, 2018), but improvements of outcomes in pain intensity and psychological disability seem to occur in all stages of pain chronification (Hüppe, Maier, Gockel, Zenz, & Frettlöh, 2011). In our analysis, we controlled for illness duration, i.e., pain chronification. However, future research might profit from further clarifying the contemporary and sequential associations between pain chronicity, incongruence and different pain dimensions.

In sum, this is the first study demonstrating that motivational incongruence plays a role in chronic pain, as it is elevated in these patients in comparison to healthy controls and is reduced by multimodal interdisciplinary pain treatment over time. Chronic pain is a disorder with both biological and psychosocial factors contributing to the development and chronification of pain (Gatchel et al., 2007). By reducing motivational incongruence in chronic pain patients, focusing specifically on approach goals, it might be possible to alleviate psychological distress in chronic pain patients. Considering that changes in pain intensity did not significantly contribute to treatment outcome, it might be a more worthwhile treatment approach to focus on improving well-being despite pain, which may be fostered by targeting chronic pain patients’ ability to satisfy their psychological needs, i.e., reducing motivational incongruence. Taken together, both clinical practice and future research may benefit from further investigations of the role of psychological need satisfaction and motivational incongruence in chronic pain treatment.
